# Test-retest reliability and concurrent validity assessment of a novel high-frequency sensor device for anterior tibial translation measurement in loaded and unloaded condition: an exploratory cross-sectional study

**DOI:** 10.1186/s12891-024-07343-y

**Published:** 2024-03-16

**Authors:** Valentin Deiss, Philippe Bähler, Pascal Kolly, Anton Schärer, Philippe Henle, Patric Eichelberger, Nathanael Lutz, Heiner Baur

**Affiliations:** 1 Department of Health Professions, Physiotherapy, Bern University of Applied Sciences, Bern, Switzerland; 2 Institute for Human Centered Engineering, Bern University of Applied Sciences, Biel, Switzerland; 3 Department of Knee Surgery and Sports Traumatology, Sonnenhof Orthopaedic Center, Bern, Switzerland

**Keywords:** Anterior tibial translation, ACL injury, Functional joint stability, High frequency sensor device, Knee kinematics, Reproducibility of results

## Abstract

**Background:**

Magnetic resonance imaging (MRI) and manual tests remain the standard for diagnosing anterior cruciate ligament (ACL) rupture. Furthermore, the passive knee displacement, also described as anterior tibial translation (ATT), is used in order to make decisions about surgery or to assess rehabilitation outcomes. Unfortunately, these manual tests are limited to passive situations, and their application to assess knee stability in loaded, weight-bearing positions are missing. Therefore, a new device with high-performance sensors and a new sensor setting was developed. The aim of this exploratory cross-sectional study was to assess the test-retest reliability of this new device in a first step and the concurrent validity in a second step.

**Methods:**

A total of 20 healthy volunteers were measured. Measurement consistency of the new device was assessed on the basis of reliability during Lachman test setting and in loaded position by artificial knee perturbation in a test-retest procedure. In a second step, the concurrent validity was evaluated with the Lachmeter® as a reference instrument. Intraclass correlation coefficient (ICC), standard error of measurement (SEM), the minimal detectable change (MDC) and Bland-Altman analysis were evaluated to assess the quality criteria.

**Results:**

The measurements with the new device during the Lachman test provided a mean ATT of 5.46±2.22mm. The SEM ranged from 0.60 to 0.69mm resulting in an MDC between 1.67 and 1.93mm for the new device. In the loaded test situation, the mean ATT was 2.11±1.20mm, with test-retest reliability also showing good correlation (*r*>0.83). The comparison of the two measurement methods with an ICC of (*r*>0.89) showed good correlation, which also underlines the reasonable agreement of the Bland-Altman analysis.

**Conclusions:**

The evaluation of the test-retest reliability of the new device during the knee stability testing in passive situation as well as in a functional, loaded situation presented good reliability. In addition, the new device demonstrated good agreement with the reference device and therefore good validity. Furthermore, the quality criteria demonstrated the ability of the new device to detect the cut-off value (3-5mm) described in the literature for the diagnosis of ACL-deficient knees, which underlines the clinical relevance of this new device as a reliable and valid tool.

## Background

The major topic of knee stability, including structural and functional stability, plays an essential role in diagnosing and rehabilitation of anterior cruciate ligament (ACL) injuries. Magnetic resonance imaging (MRI) and clinical knee stability tests are the clinical standards to evaluate ACL integrity or rupture. Manual tests like the Lachman test, Pivot Shift or Drawer Sign, which test passive knee stability, are still crucial in the examination process of supposed ACL ruptured knees. The literature shows that the Lachman test is one of the most sensitive manual tests to evaluate the function of the ACL [1, 2]. It also has excellent reliability and high validity to predict an ACL injury [2, 3]. As one of the most researched arthrometer to assess passive knee stability, the KT-1000 is considered as a "gold standard" for measuring anterior tibial translation (ATT). Schuster et al. (2004) have demonstrated in their research that the comparable and practice-orientated Rolimeter is just as reproducible and reliable as the KT-1000 [4]. In addition, it has equally good sensitivity and specificity in the assessment of ACL-ruptures [4, 5]. Furthermore, Ericsson et al. (2017) demonstrated that the Lachmeter® (www.newarthrometer.com) is a valid and reliable (*r* = 0.93 to 0.99) tool for quantifying anterior knee displacement in millimeters (mm) [6]. Therefore, the Lachman test setting is considered to be the most common reference situation and can therefore serve as a reference test for evaluation. Unfortunately, these manual clinical tests are limited to static and passive settings and do not test knee stability in functional, loaded situations, such as walking or jumping. In addition, active screening tests like hop test batteries for evaluating knee stability in a functional setting has gained in importance. However, this shift to a more active approach in testing knee stability suggests how knee function and resilience is increasingly being assessed and how active knee stability should be tested in the future. Therefore, it would be beneficial to have a reliable and valid device to test ACL patients not just in a passive test setting, but also in functional, loaded situations or activities.

Kvist & Gillquist (2001) already investigated this topic of loaded, dynamic knee stability with the CA-4000 electro goniometer in various loaded squat positions [7]. Due to soft tissue bias the measurement characteristics of the CA-4000 was found to be of low quality [8, 9]. For this reason, the Movement Laboratory of the Bern University of Applied Science developed an adjusted instrument, which has less soft tissue bias and works with high-performance sensors. As a result of the more precise sensor setting and the higher sampling frequency of the sensors, it is possible to measure with a higher measuring accuracy even in functionally, loaded situations. Consequently, the new device was developed to measure anterior tibial translation not only in an unloaded passive setting, but more importantly in a functional, loaded situation to evaluate knee stability. Therefore, the goal of the present exploratory cross-sectional study was to assess the quality criteria of this new device as a measurement instrument to determine knee stability and to measure tibial translation in a non-invasive measurement setting.

The first objective was to assess the test-retest reliability of this new device in the reference test setting situation in static position as well as in the dynamic, loaded situation as previously described by Bruhn et al. (2011) and Friemert et al. (2005) [10, 11]. The second aim of this study was to assess concurrent validity of the new device compared to the reference test setting during the Lachman test with the Lachmeter®.

## Methods

The study used a cross-sectional, exploratory design with test-retest analysis to assess the reliability of the new device, following GRRAS guideline [12]. Methodology adhered to the COSMIN Risk of Bias tool [13]. The study was appraised and approved by the local ethics committee of Bern (Cantonal Ethics Committee for Research Bern, Switzerland, Project ID: Req-2020-00613). All measurements were conducted at the Bern Movement Laboratory at the Bern University of Applied Science, Department of Health Professions. The recruitment of the subjects and the measurements took place between November 2020 and February 2021. All participants signed a written informed consent and confirmed their voluntary participation prior to the enrolment.

### Participants

The measurements were performed on a total of twenty healthy subjects (age: 29.9 ± 4.3 years; height: 172.6 ±7.1cm; weight: 67.8 ±12.4kg; women: *N* = 11, men: *N* = 9,) with no current history of disease, nor acute knee pain or injury. Further in- and exclusion criteria are listed in Table 1. A small subgroup of three out of twenty participants had ACL reconstruction older than 12 months with no symptoms or activity limitations and were therefore also included. The mean score of the Tegner activity scale was 6.3 ±0.7. The included participants frequently practiced in sport and recreational activities including stop and go activities like football, handball, tennis or winter sports like alpine skiing and ice hockey [14]. The subjects were recruited through the personal network of the author group. Due to the exploratory pilot character of this study, no a priori sample size calculation was performed [15]. The chosen sample size was based on previous exploratory studies to assess reliability or validity [6, 10, 11].

**Table 1:** In – and exclusion criteria of the present study

**Inclusion**	**Exclusion**
Aged between 18-60	Acute knee pain or injury
Healthy (no acute musculoskeletal injuries or illnes like infection)	In medical treatment cause of knee problems
Free of any sort of pain	Knee surgery within the last 12 months
Free knee mobility	Neuromuscular or vascular disease
Sufficient understanding of the German or English language	Other acute or limiting power limb/trunk injury

### Experimental protocol

After confirming their voluntary participation, demographic data (age, body height, body mass, gender, and activity level with the Tegner score) was collected. Two different test procedures (Lachman test, reflex test) with a total measurement time of about 60 min per subject were conducted. Subjects wore shorts and were barefoot for both test procedures. All measurements were conducted by the same person, an experienced physiotherapist and first author of this manuscript. All data were documented and stored in case report forms (CRF). All measurements of the 20 participants could be completed, all data conducted, and no dropouts were observed.

#### Procedure for test-retest reliability of the Lachman test setting

**Fig. 1:**
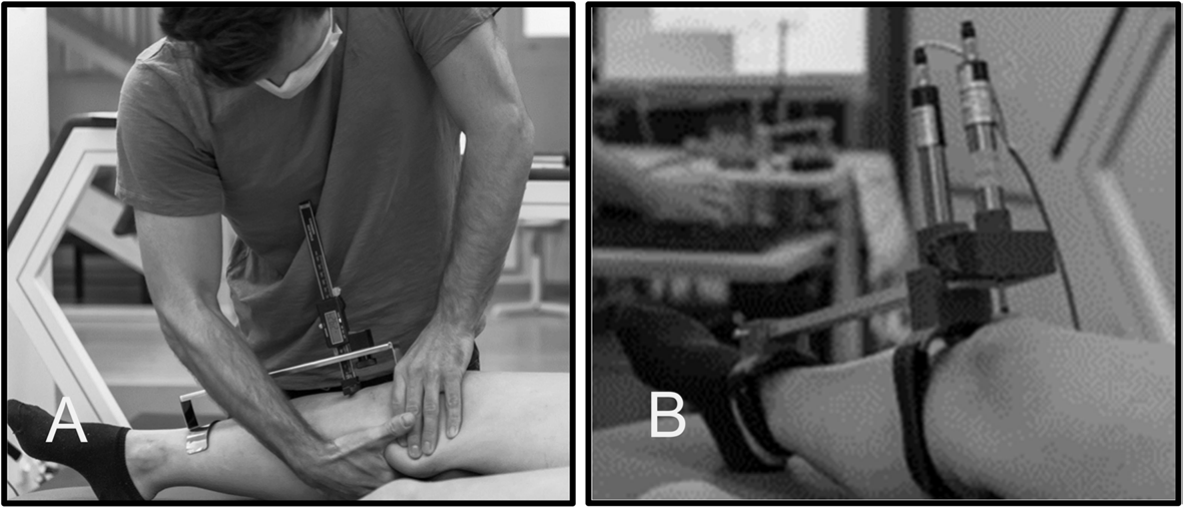
**A** the Lachmeter® and **B** the new device with the high frequent sensors setting and its closely matching design, during the Lachman test setting to assess test-retest reliability of the new device

As a first step to determine the reliability of the new measurement instrument, the test-retest reliability was assessed in the passive Lachman test setting (Fig. 1, B). As already mentioned, the Lachman test was chosen as the reference test on the basis of the literature and was used as the "gold standard" in this study for measuring anterior tibial translation in mm. Figure 1, A shows this reference test being performed with the Lachmeter® (www.lachmeter.com) (Fig. 1, A), a digital version of the well-researched, reliable and valid Rolimeter [4, 13].

Similarly, the new device closely matches this design, but features two high-performance sensors (inductive displacement sensor, measuring range: 0-20mm, type: EDCT20, MEGATRON, Putzbrunn, Germany) for measuring knee displacement. One of the sensors was positioned at the patella and the second at the tibial tuberosity as shown in the figure (Fig. 1, B). This sensor setting provides the possibility to link the measured anterior displacement of the tibia in relation to the total movement of the knee. Both sensors allow the calculation of the relative anterior knee displacement (ATT) between the patella and the tibial tuberosity during the manual Lachman test.

Participants were lying supine on the examination table and the knee relaxed in 30° of flexion, as described in the literature [6]. The centre of the tibial tuberosity was marked prior to the measurement to achieve higher accuracy and a standardised position for the placement of the two measurement instruments. Both legs were examined, the knee to be tested first was determined by randomisation (Random version 2.1.0, Volodymyr Yahenskyi). Before applying the new device to the knee, the participant had to fully extend the knee with maximal contraction of quadriceps muscle and then totally relax to achieve a standardized starting position. After adjusting the device to the knee, it was zeroed at the beginning of each assessment. The proximal hand of the examinator was placed on the knee to stabilize the position. The distal hand positioned dorsally of the distal part of the calf created the proximal anterior force for the resulting ATT [6, 14]. This anterior shear force was made with a maximum applied manual force [2, 15]. The total posterior – anterior knee displacement in the sagittal plane was recorded and presented as a total anterior tibial translation (ATT). A total of five trials per leg were performed to assess test-retest reliability.

**Procedure for test–retest reliability in loaded position**

**Fig. 2:**
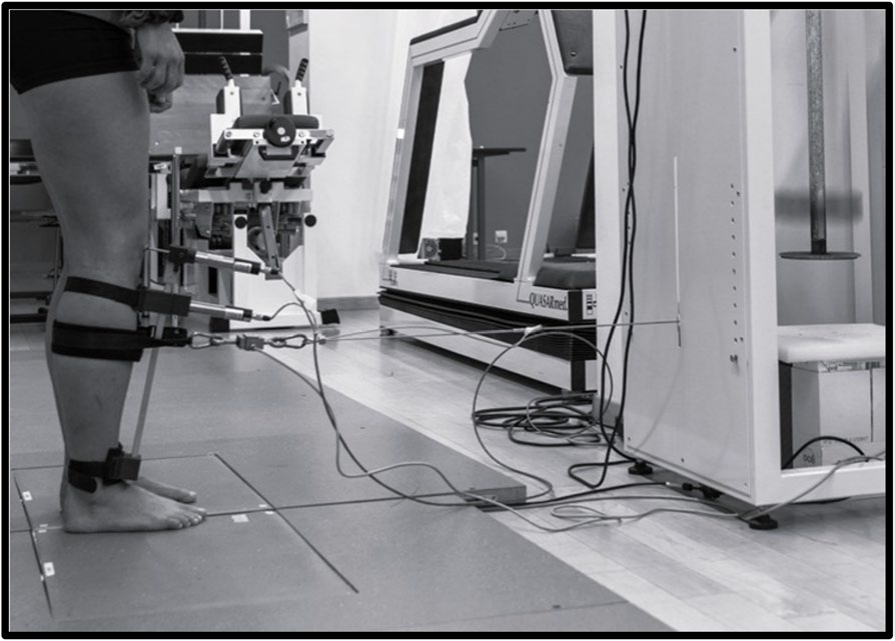
The measurement setting in loaded, bipedal standing position with the external perturbation force to assess test-retest reliability.

In order to determine data for loaded, functional setting of the new device, test-retest reliability was assessed in a loaded test setup according to Bruhn et al. (2011) and Friemert et al. (2005) [16, 17]. The subjects were standing in an upright bipedal standing position with a slight (30°) flexion of the knee (Fig. 2). An external applied force was induced to the proximal tibia, 10 cm distal of the knee joint and parallel to the tibial plateau. The mechanical perturbation was transferred by the impulse of a falling barbell weight over a rope to the tibia. This applied force pulled the tibia with an impact of 350N in a posterior-anterior direction. The impact was monitored by a force transducer (range: 0–5000 N, sensitivity 3.42 to 3.36 pC N1, linearity ±0.2 to 0.3%, Kistler, Winterthur, Switzerland), which was inserted between the pulling rope and the bandage sling around the tibia. The onset of perturbation was used as a trigger to determine the onset of the resulting anterior tibial translation by visual control. For avoiding stimulus anticipation, the participants were visually and acoustically uncoupled by wearing ear protection and visually focusing on a screen. In order to ensure an even weight distribution on both legs, the participants stood on two force plates which recorded the weight distribution.

The load on the two force plates was displayed on a screen and had to be visually checked and adhered by the participants to maintain an equilateral load.

The effect of perturbation on the displacement of the knee, the anterior translation of the tibia and the resulting total ATT was measured with the new adjusted device. Participants were tested with two series per knee, each consisting of five ventral perturbations. The first series (M1) and the second series (M2) per leg were separated by a break of 120 seconds. During this break, the position of the measuring system was checked, readjusted and the measurement data was saved. After saving all data of the first leg, the new device was fitted on the other leg to obtain data in the same way.

**Procedure to assess the concurrent validity of the new device**

As a final step, to evaluate the concurrent validity of the new device, data was collected using the reference test in the passive Lachman test setup. As already mentioned, the Lachmeter® (www.lachmeter.com) (Fig. 1, A) was used as the "gold standard" for measuring the anterior tibial translation in mm, which is the digital version of the Rolimeter [4, 13]. The test to collect the data for the concurrent validity assessment was carried out in a passive test situation in the supine position as described above in section "Procedure for test-retest reliability of the Lachman test setting" and according to Ericsson et al. (2017) [6].

Before applying the Lachmeter® to the knee, the participant had to fully extend the knee with maximal contraction of quadriceps muscle and then totally relax to achieve a standardized starting position. After adjusting the device to the knee, it was zeroed at the beginning of each assessment.

The proximal hand of the examinator was placed on top of the frame of the Lachmeter® on the patella to stabilize the device. The procedure was performed in exactly the same way as described above for the passive test-retest situation (2.2.1) with a maximum manual force applied to the proximal tibia to provoke an ATT [6, 14]. The total posterior – anterior knee displacement in the sagittal plane was recorded and presented as a total anterior tibial translation (ATT). A total of five trials per leg were performed to assess concurrent validity.

### Statistical analysis

In order to determine the quality criteria of the new measurement instrument, the test-retest reliability was assessed as the first objective to evaluate the accuracy and reproducibility of the new measurement instrument. For the purpose of calculating the reliability, the same statistical analysis was performed for both test settings, following the statistical three-layer approach according to Weir (2005) [18]. Initially, the performance of a repeated measure of the analysis of variance (ANOVA) was performed to compute in a second step the intraclass correlation (ICC). Subsequently, the standard error of measurement (SEM) was determined to finally ascertain the minimum detectable change (MDC). Additionally, the limits of agreement with its 95% CI were illustrated by a Bland & Altman plot with the mean difference of the two measurements series of the new device (d = M1 - M2) [19]. First, the ICC was used to show the consistency in the reference test setting. For this purpose, the ICC (2, 1) according to Koo & Li (2016) was chosen for two-way random effects, absolute agreement and single measurements. Second, the ICC (2,k) according to Koo & Li (2016) was chosen for two-way random effects, an absolute agreement and multiple measurements to evaluate the intraclass correlation in the loaded test setting, [20]. The ICC values were interpreted according to Koo & Li (2016) with values under 0.5 expected to show poor reliability, values between 0.5 and 0.75 showed moderate, values between 0.75 and 0.9 demonstrated good and values close to 1.00 demonstrated almost perfect reliability values [20].

The standard error of measurement (SEM) was additionally displayed to detect how far repeated measurements differ from each other. This value was calculated by the ICC and the standard deviation of the difference. For this purpose, the SEM of agreement according to De Vet et al. (2011) was then calculated and displayed [21].

$${SEM}_{agreement}= \surd ({\sigma }_{o}^{2} + {\sigma }^{2} residual)$$

This systematic error of measurements of agreement (*SEM*_*agreement*_) is thereby calculated from the square root of the sum of the error variance between observers and the residual variance *(σ *^*2*^_*o*_* + σ *^*2*^_*residual*_*)*.

$$MCD=SEM*1.96* \sqrt{2}$$

Furthermore, minimal detectable change (MDC), representing the sensitivity to change, was calculated, using the SEM, with the formula according to Ries et al. (2009) [22]. Additionally, the limits of agreement with its 95% CI were illustrated by a Bland & Altman plot with the mean difference of the two measurements series of the new device (d = M1 - M2) [19].

To determine the concurrent validity, repeated measurements on the Lachmeter® were calculated with the intraclass correlation coefficient (ICC) and further used to calculate the agreement between two measurement devices with the corresponding five measurements per test device and knee in the static, passive Lachman test setting. For this purpose, the ICC [2, 1] according to Koo & Li (2016) was chosen for two-way random effects, absolute agreement and single measurements [20]. The standard error of measurement (SEM) was additionally displayed to determine how much the two methods differ from each other. The MDC was also used as already illustrated in the formula earlier, representing the sensitivity to change. As a further step, the agreement between the two methods was analysed using Bland & Altman (B&A) analysis with the calculation of the systematic bias and the limits of agreement (LOA) with its 95% confidence interval (CI) [19].

For statistical calculations the RStudio software package was used (RStudio Version 2022.07.1 +554; RStudio, PBC, at 250 Northern Avenue, Suite 410, Boston, Massachusetts 02210).

## Results

All results are presented as mean ± standard deviation (SD). The measurement results of the passive, manual test situation ranged from 0.83-13.03mm with the new device, while the mean values of the measured anterior tibial translation were 5.46±2.22mm. The small subgroup of the three participants with ACL reconstruction, of the total 20 participants measured, presented a higher mean anterior knee displacement, with a higher SD value and were partly responsible for the overall higher mean value and SD. The mean ATT of the three ACL reconstructed knees was 8.5±3.7mm with the new device. The dynamic test setting in loaded, standing position showed an overall mean over the two series (M1 and M2) of 2.11±1.11mm.

The Lachmeter® measurement in the passive, manual test situation ranged from 1.22–11.28mm with a mean anterior tibial translation of 5.27±2.04mm. The small ACL subgroup with the three ACL reconstructed knees showed a mean ATT of 8.8 ±2.0mm.

### Descriptive statistic

**Fig. 3:**
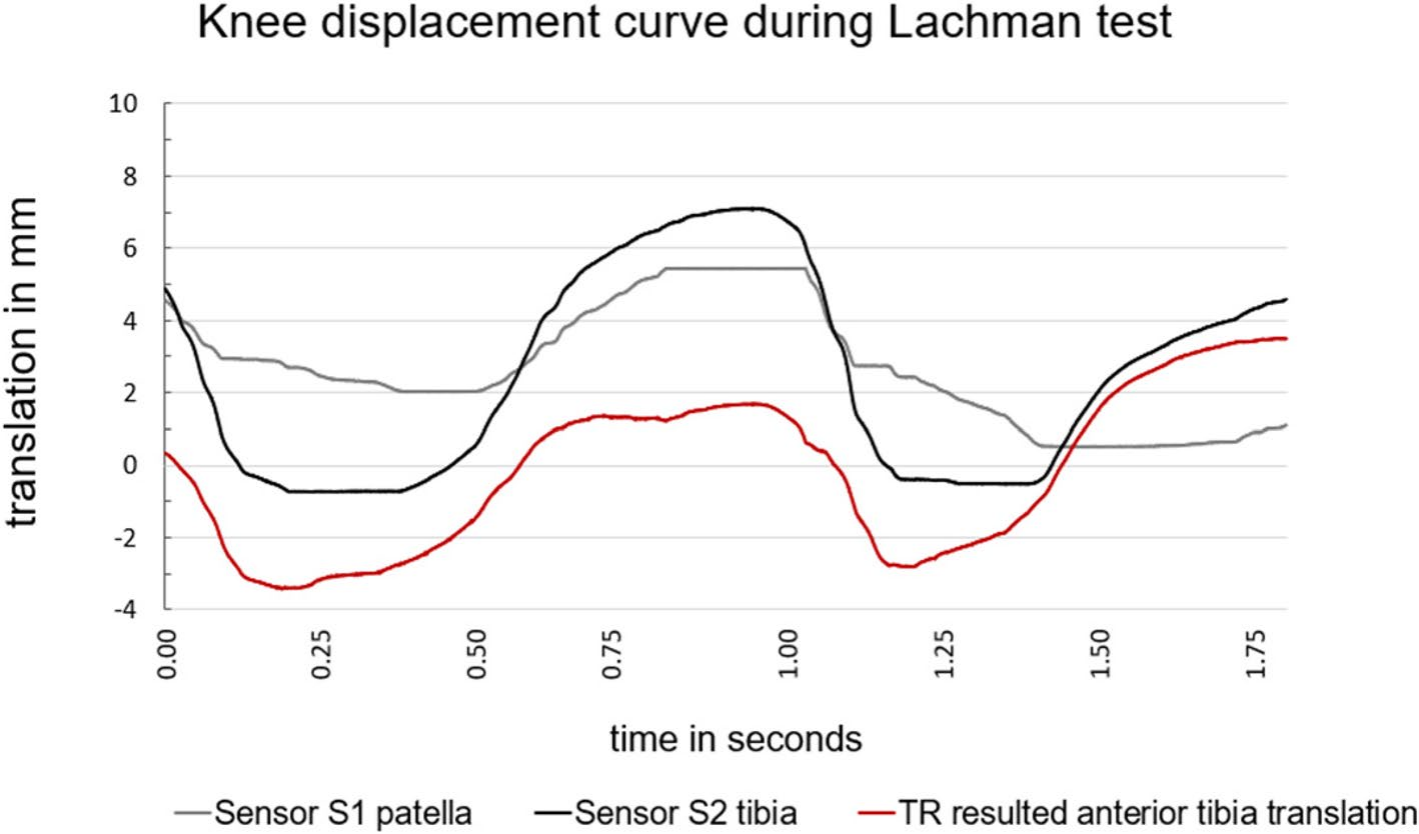
The diagram shows the motion-time curve of the static, passive measurement during the Lachman test setting with the new device. The grey line shows the patella sensor S1, the black line shows the tibia sensor S2, and red line shows the resulting total ATT (in mm).

For descriptive statistics, motion-time curve diagrams were presented for the new device, for both, reliability, and validity assessments. In order to visualize the curve progression of the static, passive Lachman test setting, Fig. 3 presents the motion – time curve diagram of the ATT, measured with the new device, with the movement amplitudes in millimetres (mm) over time in seconds (s). This figure (Fig. 3) shows a representative motion curve with an average motion deflection of a measured knee during the Lachman test with the new device.

The grey line displays the curve of the reference sensor on the patella (S1) while the black line shows the sensor on the tuberosity tibiae (S2). The red indicates the resulting total anterior tibial translation (TR), which describes the difference between S1 and S2. The maximum ATT was measured from the onset of the anterior tibia displacement to the highest value of the plateau.

**Fig. 4:**
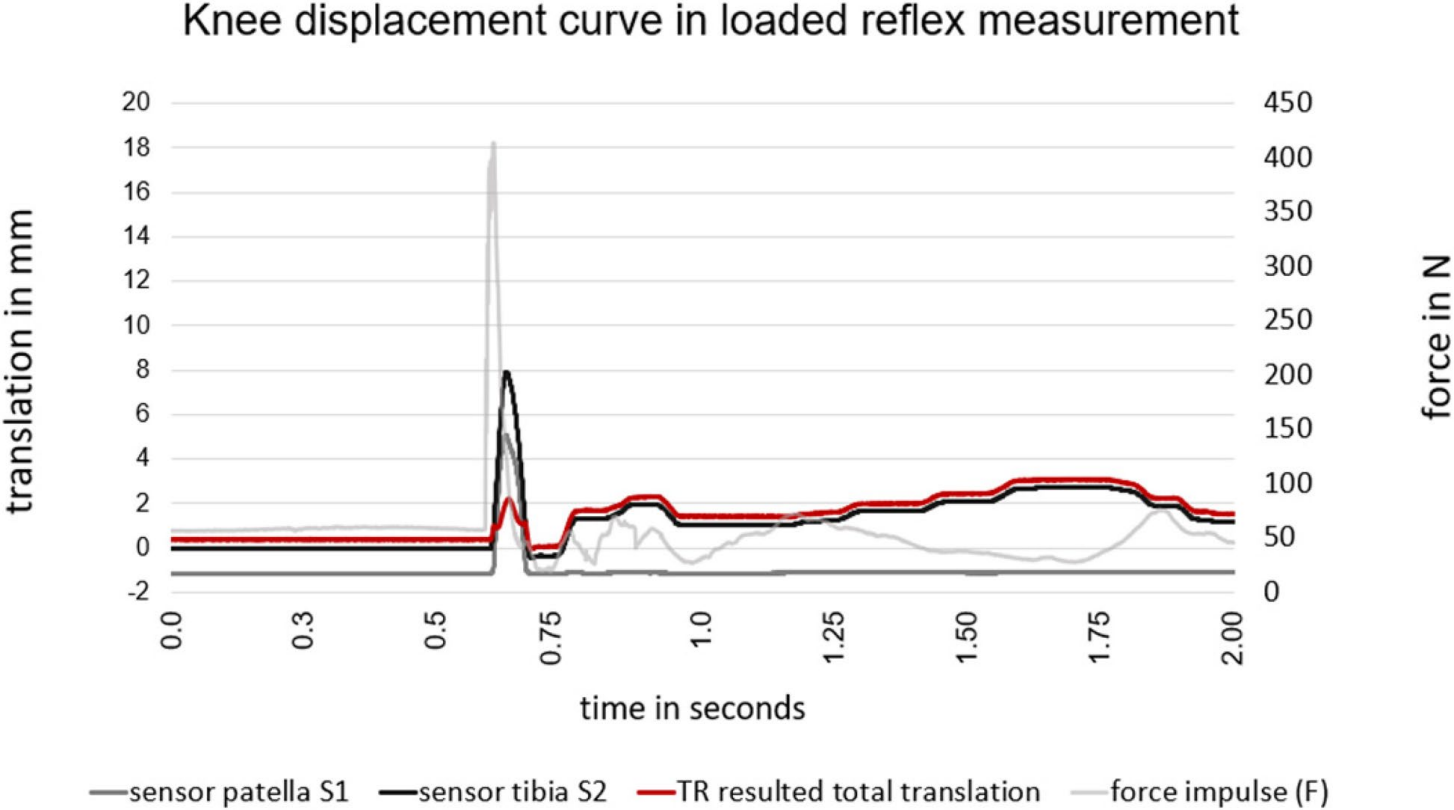
The motion-time curve of the measurement to assess the test-retest reliability illustrates the reflex curve with the resulting (TR) anterior tibial translation (ATT) of 2.2mm. The bright grey curve illustrates the perturbation impulse as a force in newton (N) and red curve shows the resulting total ATT (in mm).

A representative motion – time curve of the dynamic, loaded test situation of the reflex assessment is illustrated in Fig. 4. Equal to the static setting the sensor S1 *(grey)* recorded the anterior movement of the knee at the patella, whereas the sensor S2 *(black)* on the tuberosity tibiae recorded the anterior displacement of the tibia.

As a result, the difference of these two sensors (S1-S2) is displayed with the *red* line as the total ATT (TR).

In addition to the passive Lachman test setting, the force sensor *(F)* measured the perturbation impulse in newtons. The amplitude of the perturbation force with a mean of 350 ± 70N provoked the anterior knee displacement at the proximal tibia.

The diagram also illustrates further oscillations of the two sensors after the actual perturbation impulse. These additional oscillations were inter alia caused form swinging of the rope, but mainly of repositioning of the participant. The peak of the total ATT generally occurred approximately 40ms (± 5ms) after perturbation impulse.

### Test-retest reliability of the Lachman test

For assessing the test-retest reliability of the new device in passive Lachman test setting, ICCs of the repeated measurements were first compared according to the mentioned three-layered approach [18]. Table 2 contains the different ICCs for each test setting, the device, and the knee side to assess test-retest reliability.

**Table 2:** The table shows the Intraclass correlations coefficients (ICC) and the standard errors of measurements (SEM) of the repeated measurements during the Lachman test setting and the reflex measurement.

	**Lachman test setting**				**Reflex measurement**	
	Lachmeter analog		new device		new device	
	*left knee*	*right knee*	*left knee*	*right knee*	*left knee*	*right knee*
ICC 2.1	0.855	0.868	0.938	0.896		
ICC 2.k					0.860	0.820
					0.833	
SEM_agreement_	0.872	0.563	0.604	0.698	0.564	0.712
					0.642	

The repeated measurements of the new device showed a measurement consistency an

ICC of 0.94 for the left and 0.90 for the right knee (see Table 2).

These values showed good intraclass correlation, interpreted according to Koo & Li (2016) [20]. Through the formula mentioned above, a SEM of 0.60mm and 0.69mm was calculated with the new device, for the left and right knee, respectively. Based on this SEM, a minimal detectable change of the new device of 1.67mm for the left knee and 1.96mm for the right knee was determined.

### Test-retest reliability in loaded position

Data collection to evaluate measurement consistency through test-retest reliability during the functional, loaded setting, resulted in 2.15 ±1.23mm and 2.08 ±1.18mm for the first (M1) and second (M2) series of measurements, respectively. According to Koo & Li (2016), intraclass correlation between the two measurement series indicated good reliability (*r* > 0.83) [20].

**Fig. 5:**
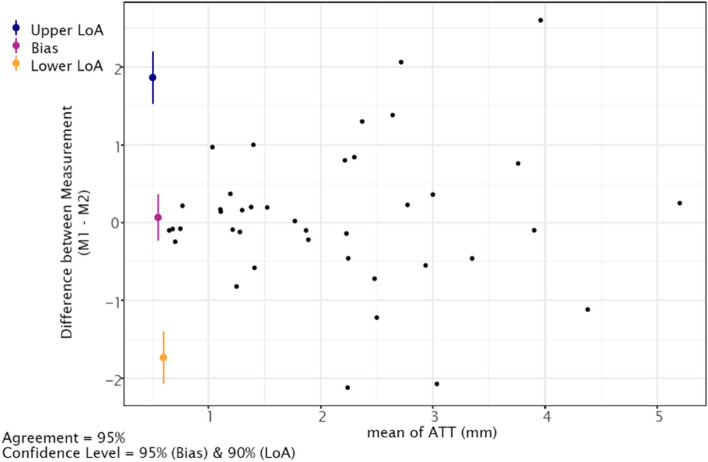
Bland & Altmann plot shows the mean difference of the Lachmeter® and the new device during the Lachman test setting. The systematic bias of - 0.18 mm (95% CI -0.71, 0.34) marked as purple line, whereas the blue and yellow lines show the limits of agreement (LOA) and 95% CI of the upper 3.01 mm and lower - 3.38 mm limits (± 1.96 SD), respectively

The calculated SEM lay at 0.64mm and thus resulted in an MDC of 1.77mm. In addition, the Bland & Altman plot (Fig. 5) calculated with the mean differences showed reasonable agreement between the two measurements (M1, M2).

The plot displays the distribution of the mean difference and the mean values of the two-measurement series. The blue signs illustrates the upper 1.86mm (95% CI 1.53, 2.19) limit of agreement, while the yellow sign shows the lower -1.73 (95% CI -2.06, -1.40) limit of agreement with its 95% CI. The systematic bias of the two reflex measurements resulted in 0.06mm (95% CI -0.23, 0.36) and is illustrated as a purple sign in the figure.

Additionally, this plot displays that values about 2mm above the mean still show high measurement agreement. Further, the three statistical outliers in the figure are random errors*.*

### Concurrent validity of the new device

For assessing concurrent validity of the new device in passive Lachman test setting, ICCs of the repeated measurements were compared (Table 2). The repeated measurements on the Lachmeter® showed a measurement consistency with an ICC of 0.86 for the left and 0.87 of the right knees. These values showed good intraclass correlation, interpreted according to Koo & Li (2016) [20].

**Fig. 6:**
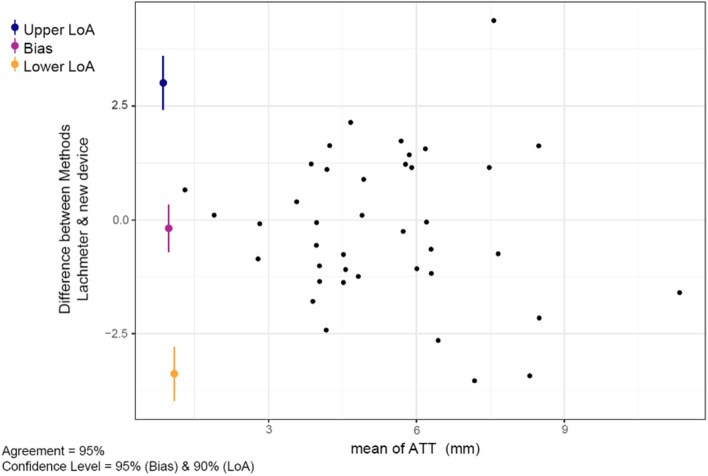
Bland & Altmann plot shows the mean difference of the first (M1) and the second (M2) measurement series. The systematic bias of 0.06mm (95% CI -0.23, 0.36) marked as purple sign, whereas the blue and yellow sign illustrates the limits of agreement and 95% CI of the upper 1.86 mm and the lower -1.73mm limit (± 1.96 SD)

Furthermore, the Bland & Altman plot (Fig. 6), calculated with the mean differences of the two methods, showed a systematic bias remained at -0.18 mm (95% CI -0.71 to 0.34), whereby the LOA ranged between from -3.38mm (95% CI -3.97, -2.79) to 3.01mm (95% CI 2.42, 3.59).

## Discussion

The objective of the present study was to assess the quality criteria of the new device for anterior knee displacement measurement in the well researched, passive Lachman test setting but also in the standardized, loaded test setting [6, 16]. This load situation was chosen for data collection and the evaluation of a more functional test set-up in standing position, as similar as possible to everyday life or sports loads on the knee joint as well as the ACL.

The first aim was to evaluate test – retest reliability of this new device in the passive, but also in the more functional, loaded test setting. The second aim was to assess the concurrent validity of the new device during the passive Lachman test setting compared to the reference measurement instrument of the Lachmeter®.

The present results from data collection during the passive manual test setting showed comparable values to those in other studies. In a previous work to validate the Rolimeter by Ganko et al. (2000), values of 10.6 ±3.0 mm were measured in ACL-injured subjects and 5.1 ±1.3mm in healthy subjects [23]. Ericsson et al. (2017), showed a smaller range of 4-10mm from the test – retest reliability study of the Rolimeter [6]. These values are in line with the values of the present study, while the new device showed a wider measurement range and confirm the clinical relevance of its use. Furthermore, Ericsson et al. (2017) proved in his study that the Rolimeter is a reliable (*r*>0.93) and a valid tool for detecting ACL injuries. In reaching equivalent values, this supports the results of the current study and confirms the clinical applicability of the new device as a consistent, reliable (*r*>0.89).

Furthermore, participant’s characteristics during the passive test setting showed higher anterior knee displacement of the small subsample of the three ACL reconstructed subjects compared to the ACL intact knees. These findings go together with the results of previous literature in assessing anterior knee displacement. Sonesson & Kvist (2017) for example measured 9.1 ±1.0mm of ACL reconstructed knees 2 to 5 years after surgery versus 7.0 ±1.7mm of healthy knees [24]. These similar mean values of ACL reconstructed knees support the differences found overall and particularly in the small ACL subsample. Moreover, these subgroup findings also show a certain heterogeneity of the present cohort. Such possibilities in measuring a wide range of ATT also allow to determine ACL insufficient or hypermobile knees. In addition, these measurement possibilities also show the practical suitability of this new device for passive testing but also in possible diagnosis of ACL-deficient knees. Therefore, these results point to a reasonable use of this new device. However, to further evaluate the clinical relevance, the next research step should include the assessment of ACL-insufficient or hypermobile knees.

In relation to the passive, manual test situation, the results to assess test-retest reliability in a functional, loaded setting, showed considerably smaller ATT values. The difference between the two test situation showed a difference of about 3.5mm. Previous literature assessing ATT in both situations (unloaded, loaded) found no correlation in passive, manual testing and functional, loaded testing [6, 25],[26]. A possible explanation for this difference in these two conditions might be the general higher muscle activation in loaded, standing position as well as the muscular pre-activation combined with joint stiffness compared to the relaxed passive Lachman test setting. Furthermore, the repeated measures in the functional, loaded test situation between the first (M1) and the second (M2) measurement showed good measurement consistency and agreement and thus good test-retest reliability. This measurement setting in a loaded position to assess reliability was performed to provide baseline data for further investigations in this functional situation (e.g., comparison of deficient leg and contralateral leg).

Only few previous studies evaluated anterior knee displacement in a functional loaded position [6, 7, 24, 26]. Kvist & Gillquist (2001) measured with the CA-4000 the max ATT in a comparable loaded bipedal squat position [7]. Their findings showed max ATT of 5.9 ±2.1mm in healthy subjects, which is noticeably higher than the measured mean of 2.1 ±1.1mm in this present experimental study. A possible explanation for the lower ATT values could be the more accurate sensor setting with less soft tissue distortion and the additional sensor at the patella of the present device. Furthermore, quality criteria of the CA-4000 from these mentioned studies were missing. This does not allow clear comparison with the current data since accuracy and the variability of the CA-4000 measurements have not been reported.

Friemert el at. (2010) also investigated anterior knee displacement in the same functional, loaded setting in healthy subject. After an equivalent anterior perturbation at the tibia of 300 N, and a mean ATT of 6.6 ±1.4mm was measured [27]. He described a plateau effect of ATT after 100-150ms, which could not be found in the current study. The explanation for this difference could be the muscular reflex response, which normally occurs around 40ms in healthy subjects. This deviation, can be attributed to the reduced reflex response, which was attempted to be suppressed in the above mentioned study [27]. This suppressed reflex response, in turn, could also explain the higher mean ATT values compared to the current study. In addition, the sensor setting was described inaccurately, which contain a certain potential bias and could also be responsible for the higher deviation. Further investigations are needed also to test the scope of the new device also in pathologic cohorts and to assess more data in functional, loaded situation to verify these findings of the present exploratory study setting.

Schuster et al. (2004) evaluated the Rolimeter in comparison to the KT1000 and showed a similar confidence interval value (2.3mm) as in the present study [4]. This similar range of CIs indicates that the data collected suggest a similarly performance of the new device and is therefore comparable with existing devices and literature. Furthermore, the MDC between 1.67mm and 1.93mm (left and right knee) calculated by the SEM indicates that smaller values cannot be interpreted as a true change and therefore no conclusion can be drawn from smaller values. Despite that, the present MDC is smaller than the often described cut off value of 3-5mm anterior knee displacement to detect an ACL deficient knee [5, 6, 25]. The literature described that mean side-to-side difference is of importance to identify a possible ACL deficient knee. Previous research has confirmed that this mean side-to-side difference was significantly greater for complete ACL tears compared to intact knees. Panisset et al (2012) has demonstrated on clinical testing that gross laxity and anterior tibial translation greater than 5 mm were significantly associated with complete ACL tears [5]. The same was confirmed by Dejour et al. (2013) in their study [25]. While the results of Ericsson et al. (2017) were already significant at a side-to-side difference of 3 mm, which was slightly below these mentioned value of 5 mm [5, 6, 25]. It is therefore possible that the new device can provide this valuable minimal clinically important difference (MCID) of the 3-5mm to detect ACL ruptures with the given MDC. Moreover, the more conservative approach of the Bland & Altman analysis using the LOA (– 3.38mm, 3.01mm) confirmed this finding for the detection of ACL deficient knees. Based on the findings, it indicates the new device to be both a reliable and valid instrument as well as a viable device for clinical use to measure anterior tibial translation during the Lachman test. Consequently, it also indicates achieving the given clinical significance by reaching the mentioned MCID. In a further step, it is shown that the new device also provides consistent and reliable measurement data in a functional, loaded situation.

The evaluation of the concurrent validity of the new device demonstrated good agreement with the reference device of the Lachmeter® in the measurement of ATT during the passive, manual Lachman test situation. Furthermore, the statistical analysis showed no significant differences between the two measurement methods, while the ICC also demonstrated a good correlation between the two. In addition, the Bland & Altman plot also presented reasonable agreement between the two methods with the LOA and the negligible small systematic bias [28]. Due to the negligible amount of systematic bias, the new device presented to be congruent with the used reference device and therefore to be a valid measurement tool.

A few limitations existed that should be considered when interpreting the results of the present study. First, this study was conducted on healthy and active individuals which could affect generalization of the results. Moreover, the exploratory nature of the current study was based on a small sample size. Based on these facts, possible differences in the resulting ATT are to be expected with a larger sample size and a patient population in a functional, loaded test situation. Furthermore, the participant cohort showed a heterogeneous character due to the incidental findings of the ACL-reconstructed subsample and displayed possibilities to capture higher knee displacement differences.

In addition to that and as a consequence of the exploratory character of the present study, the current construction showed also some limitations of the new sensor setting. One of the main problems was the size of the sensors, which leads to long lever arms and thus to susceptibility to acceleration due to their mass and the resulting inertia. Nevertheless, functional tasks such as active knee extension, sit to stand or squat position should also be assessable. Moreover, the current setup is tied to cables, which makes the device less practical. In order to have an applicable and more practicable device for clinical use and thus be able to collect data during the intended functional test, the sensors should be smaller, and the data transmission has to be wireless. Based on these facts, further adjustments and investigations must be carried out with the new device. Thus, the practical suitability and the scope of application should be further tested, and in addition, other potential limitations should be evaluated. In conclusion, the current tool offers limited applicability in the clinical setting, which affects the actual transfer to clinical situations.

Overall, the presented results are in line with previous literature that measured ATT in a passive manual Lachman test situation and proves the new device to be reliable and valid. In addition, the present study provides good consistency and reproducibility in the functional, loaded test situation for the test-retest measurement of the new device. The results demonstrated lower, but explainable deviations in the measured values, compared to data from studies with similar test set-ups. Nevertheless, these results add essential data to the measurement of anterior knee displacement in a loaded position in the framework of investigating knee stability. Further studies are needed to test additional clinically relevant functional situations and activities to assess knee stability with their ATT in these loaded situations.

Despite these promising results, mentioned questions like practicability, clinical applicability and transfer to other loaded settings remain. Further work is required to establish the suitability of such a new device for clinical use.

## Conclusion

The result of this explorative cross-sectional study showed the new device to be a reliable and valid measurement instrument to assess anterior knee displacement. Furthermore, the data collected in the loaded, standing position for the assessment of consistency showed good correlation and reasonable agreement of the test-retest evaluation. These present findings of the new device offer further possibilities in assessing knee stability in dynamic and loaded situations. It also provides the opportunity to test ACL-deficient knees for decision making on ACL reconstruction or as a test criterion during ACL rehabilitation. Further research is required to evaluate other clinically relevant functional, loaded test settings of this tool in more dynamic situations like walking, stairs climbing, squat or even jump settings. Additional studies will be needed, which also investigate in ACL-deficient or hypermobile knees to gain more insight into the variability of anterior tibial translation in different cohorts. These findings will help adding useful data to clinical decision making in the future.

## Data Availability

The datasets generated and/or analysed during the current study are not publicly available for data protection reasons. Data is available from the corresponding author on reasonable request.

## Abbreviations

ACL: Anterior cruciate ligament
ATT: Anterior tibial translation
ICC: Intraclass correlation
LOA: Limits of agreement
MCID: Minimal clinical important difference
MDC: Minimal detectable change
MRI: Magnetic resonance imaging
SEM: Standard error of measurement
TR: Total anterior tibial translation
